# A modified Delphi study to identify screening items to assess neglected sexual side-effects following prostate cancer treatment

**DOI:** 10.1186/s12894-022-00982-0

**Published:** 2022-03-11

**Authors:** Pierre Röscher, Kimesh Naidoo, Joanne E. Milios, Jacqueline M. van Wyk

**Affiliations:** 1 Nelson R. Mandela School of Medicine, University of KwaZulu-Natal, 719 Umbilo Rd, Umbilo, Berea, 4001 South Africa; 2 Clinical Head of Unit: Paediatrics, King Edward VIII Hospital, and Clinical Researcher Nelson R. Mandela School of Medicine, University of KwaZulu-Natal, 719 Umbilo Rd, Umbilo, Berea, 4001 South Africa; 3 Clinical Researcher and Professional Practice, School of Sport Science, Exercise & Health, The University of Western Australia, Parkway Rd, Crawley, 6009 Australia; 4 Clinical and Professional Practice, Nelson R. Mandela School of Medicine, University of KwaZulu-Natal, 719 Umbilo Rd, Umbilo, Berea, 4001 South Africa

## Abstract

**Background:**

Neglected sexual side effects (NSSE) are a group of less common sexual side effects that may present after Prostate Cancer (PCa) treatment. There is currently no valid and reliable tool to identify these side effects. A modified Delphi study is an effective way of developing the content of such a screening tool.

**Methods:**

A modified Delphi study was used to obtain consensus from a multi-disciplinary group of experts over 3 rounds during a 12 week period. Ten statements were presented containing 8 closed-ended statements on individual NSSEs, and 2 open-ended statements on psychosocial impact related to NSSE. Consensus was defined as a 75% strongly agree achievement on each statement, or the final statement evolution at the end of 3 rounds. Statement support in each round was determined by mean, standard deviation and range, after a numerical value was allocated to each statement during specific rounds. All three rounds were structured and suggestions and additions were incorporated in the statement evolution of the three rounds.

**Results:**

Thirty-five participants were invited, and 27 completed Round 1 (RD 1), 23 participants completed RD2, and 20 participants completed RD3. All 3 rounds were completed in 12 weeks. Statement 1 (sexual arousal incontinence), statement 2 (climacturia) and statement 3 (orgasm intensity) reached consensus after RD2, and statement 9 (sexual dysfunction impact) and statement 10 (experiences) were removed after RD3. Statement 4 (orgasmic pain), statement 5 (anejaculation), statement 6 (sensory disturbances), statement 7 (penile length shortening) and statement 8 (penile curvature) were finalised after the conclusion of RD3. Statements 1–3 were the most stable statements with the most support and least amount of disagreement. Statements 4–8 were less stable, but support for them improved over the 3 rounds. Statements 9–10 both had good stability, but the support indicated that they needed to be removed from the set of statements. Statement 5 had the poorest range due to an outlier opinion.

**Conclusions:**

Consensus was reached on the items making up the NSSE screening tool. Health care practitioners will be able to use this tool to identify the evidence of NSSE after PCa treatment. Further testing will be undertaken to confirm the reliability and validly of the tool.

**Supplementary Information:**

The online version contains supplementary material available at 10.1186/s12894-022-00982-0.

## Background

Disability amongst men related to sexual dysfunction is high following their diagnosis and treatment for prostate cancer (PCa) [1, 2]. The reported incidence of PCa globally was 1.3 million cases in 2017, but more importantly, this was responsible for 7.1 million disability adjusted life years in these diagnosed men [3]. The average age of PCa diagnosis in South Africa is 68 years, and the average age of death due to PCa is 74 years [4]. The risk of developing PCa increases exponentially after the age of 50 years for South African men, and older age and ethnicity (African black men) are the most notable non-modifiable risk factors leading to more aggressive PCa [5]. Treatment of localized PCa may include surgical (radical prostatectomy) and non-surgical interventions (radiation therapy) amongst others [6]. These interventions may cause disabling side effects that may include pain, incontinence and sexual dysfunction [2, 7–9]. Only 20% of men will reportedly ever discuss issues of sexual dysfunction with their health care practitioner after PCa [10] and while they may recover from pain and incontinence, they will suffer debilitating and long-lasting effects because their sexual dysfunction remained undetected [1, 11].

The less common symptoms of sexual dysfunction after PCa treatment may present in the form of a variety of complications that are collectively referred to as “Neglected Sexual Side Effects (NSSE)” [12, 13]. These NSSE drastically impacts the quality of life in many men, as their urinary, sexual, bowel and hormone functions may be already adversely affected, creating additional daily challenges for them [9]. NSSE range from anejaculation, change in penile length and curvature, urinary incontinence during sexual activity (climacturia), arousal incontinence, orgasmic disturbances that encompass anorgasmia, changes in orgasmic sensation and painful orgasm among others [12, 14]. Sexual function is the quality indicator most strongly associated with outcome satisfaction after PCa treatment [9] and sexual dysfunction is a predictor of bother and depression after PCa treatment [15, 16]. Poor sexual function has been associated with a higher prevalence and severity of depressive symptoms, and these symptoms may have a lasting psychological impact after the diagnosis of PCa [17].

A literature review indicated a few original publications on the NSSEs after PCa treatment, but only two publications address issues on how to assess the NSSEs [12, 14]. Both studies used an informal non-validated outcome measure to determine the extent of the NSSE. Other common PCa related sexual dysfunction outcome measures includes the Expanded Prostate Cancer Index Composite (EPIC) [18] and the International Index of Erectile Function (IIEF) [19]. The EPIC and IIEF are both validated instruments and both were recommended at the Fourth International Consultation for Sexual Medicine in 2015 [11]. However, both instruments only address general sexual dysfunction and there are no questionnaires to assist in diagnosing the NSSEs after PCa. There was thus a need to develop and validate an instrument that will effectively confirm the evidence of the NSSE after PCa treatment.

The aim of this study was to bring together a group of experts to develop an instrument that could be used as a self-administered clinical screening tool to identify NSSE 1–10 years after PCa treatment. A Delphi technique study provides such an opportunity where experts can give controlled feedback to develop a group opinion on a specific subject [20]. The Delphi technique has proven to be a reliable measurement instrument to develop and to refine a new concept, and to direct future research [21]. The Delphi technique is also a cost effective and efficient method to collect information from an expert panel of participants, and is ideally suited for electronic administration [22].

The study explored the questions that should be included in a screening tool to investigate the NSSE after PCa and it sought gather consensus on the appropriate wording of statements from a group of experts to include in the NSSE screening tool.

## Methods

### Study design

A modified Delphi study was performed according to the methodological criteria of Diamond et al. [20]. The Delphi technique was used to obtain consensus among experts on the questions to include in a screening tool for NSSE after PCa treatment, where patients would be asked to indicate their experienced NSSE symptoms relating to the previous 3 months. Three rounds of the study survey were circulated [23]. The participants were recruited via email and a Google Forms link was provided for their participation. The duration of the study was predetermined [20], and was set as 3 rounds each consisting of 3 weeks, with a one-week collation time after each round, making the total duration of the study 12 weeks. The time to complete each round was suggested to take only 10–15 min. Participants were assured anonymity and informed of their right to withdraw at any time. All the participating experts gave informed consent to participate in the study. Consensus was defined, and the termination of the study was described. Each participant was asked to complete the study survey independently and were given instructions on how to complete each round of the study. The original research statements that were used in round 1 can be found in Additional file 1: Appendix 1.

The first round (RD 1) collected demographic information from the expert panel. All three rounds (RD1-3) presented a set of statements in the form of questions to be posed to a potential patient. The experts were asked to indicate how appropriate they thought the statement was by ranking it on a 5-point Likert scale (“strongly agree, agree, neutral, disagree and strongly disagree”) and they were asked to comment on each statement. This allowed for the identification of statements that were unclear or required additional attention. Once a participant submitted their survey answers, the study moderator was able to collate their information and code each participants’ data into an Excel spreadsheet. Participants who had not yet responded during each round were received two additional reminders to complete the round, and the Google form link was closed after three weeks. The research team discussed and implemented all the comments and suggestions and communicated the changes and the new version of the screening tool to the experts during subsequent rounds. The experts were thus asked to rank the appropriateness of a new set of statements in RD2 an RD3 according to the changes that the collective group of experts requested in the previous rounds.

### Data analysis

Quantitative and qualitative data was produced in all three rounds of this study. The quantitative data was represented by the percentage of participants choosing the “strongly agree option on the Likert scale, as we aimed to achieve a 75% approval rating in each round. In addition to this, RD 1 produced quantitative demographic data. The qualitative data was represented by the comments and the suggestions submitted by the experts in each round. A deductive approach was used to code the comments and suggestions (the perceptions of the participants) into a specific framework [24]. This framework included the directional views of the experts (positive/negative/indifferent) and were applied by the authors where these themes matched the theory regarding the NSSE after PCa treatment. This data dictated the changes made to the statements in each subsequent round.

### Expert panel

We identified a group of multi-disciplinary medical experts working in the field of prostate cancer and sexual medicine in South Africa. An additional international (Netherlands) expert (medical sexologist) was identified from outside the setting due to the small number of appropriately qualified medical sexologists practicing in South Africa. In addition to working in the prostate cancer field, the overwhelming majority of the identified experts were either members of the South African Sexual Health Association or where affiliated to the Prostate Cancer Foundation of South Africa. Thirty-five potential participants were invited via email to participate in this study. This multi-disciplinary group consisted of urologists in the field of radical prostatectomies, urologists in the field of prostate radiation therapy, oncologists, medical sexologists, psycho-sexologists, psychologists, and pelvic health physiotherapists. There is no set participant number needed to conduct a Delphi study in the literature, but most Delphi studies usually use between 15 and 20 participants [25, 26].

### Consensus criteria

Consensus was predetermined as one of two scenarios. In scenario one consensus was defined as a 75% agreement/or disagreement amongst the participants on each questionnaire statement description on the final option of the 5-point Likert scale [27], in this case “strongly agree that the statement is appropriate”. In cases where scenario 1 was not achieved, scenario 2 would be actioned. Consensus via scenario 2 was defined as the majority agreement of statements after the three-round process where consensus was not previously reached [20].

### Statement support

The support of the statement between panellists in each round were determined by the mean, standard deviation and the range of each statement. A numerical value was matched with each Likert scale answer as follows: Strongly Agree = 1, Agree = 2, Disagree = 3 and Strongly Disagree = 5. The ideal mean would be 1, meaning that all the participants strongly agreed on a specific statement. A smaller standard deviation meant a bigger convergence towards strongly agree within a round, and a smaller range in each round meant a more unified opinion between experts within a round.

### Questionnaire content

The content covered in the questions circulated in RD 1 was derived from available literature on the NSSE after radiation therapy for PCa [12] and on NSSE after a prostatectomy [13, 14]. The questionnaire consisted of eight specific questions relating to each of the NSSE after PCa and a matching 5-point Likert scale for each question, and 2 open-ended questions on the psychosocial impact of having and dealing with PCa.

## Results

This section presents the results of the Delphi study and how consensus and stability evolved over the RD1-RD3 by looking at:

- The composition of the expert panel
- Agreement percentages
- The evolution of statements
- The support of statements by the expert panel.

**Fig. 1:**
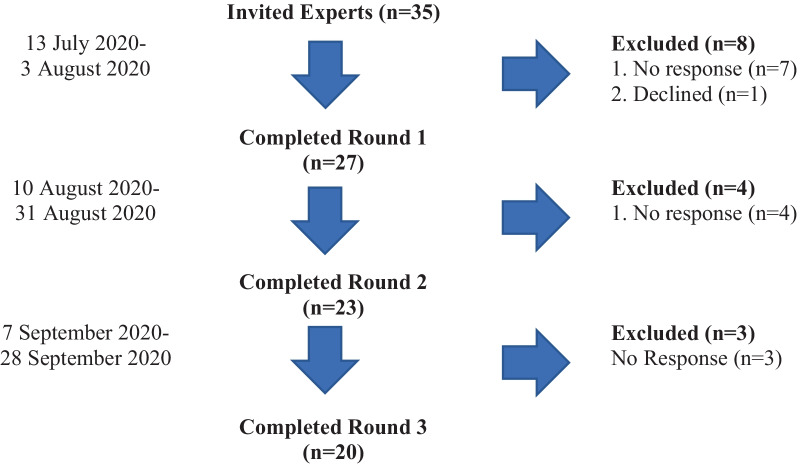
The modified Delphi technique rounds 1–2

Thirty-five participants were initially invited to participate in the study, and 27 responded and completed round one, 23 participants responded and completed round two, and 20 participants completed round three (Fig. 1). The 3 rounds were successfully executed in the planned 12-week time frame.

### Participant demographics: RD 1–3

Table 1 describes the detailed demographic information of the participants during each round. The largest professional representation of the experts during round 1 were the combined group of urologists (30%), followed by the combined group of sexologists (26%) followed by the physiotherapists (22%). This trend remained consistent during round 2 except for the sexologists making up the majority of the group at 30%. This was similar in round 3 with the sexologists constituting 30% of the expert group and the urologists and physiotherapists each representing 25%.

**Table 1:** Participant demographics round 1–3

Participant demographics	Round 1	Round 2	Round 3
Number	27	23	20
*Age*			
31–40	5 (19%)	3 (13%)	2 (10%)
41–50	11 (41%)	9 (39%)	8 (40%)
51–60	8 (30%	8 (35%)	8 (40%)
> 61	3 (11%)	3 (13%)	2 (10%)
*Gender*			
Male	13 (48%)	11 (48%)	9 (45%)
Female	14 (52%)	12 (52%)	11 (55%)
*Profession*			
Oncologist	4 (15%)	3 (13%)	2 (10%)
Physiotherapist	6 (22%)	5 (22%)	5 (25%)
Psychologist	2 (7%)	2 (9%)	2 (10%)
Sexologist (with a medical background i.e. a GP)	3 (11%)	3 (13%)	2 (10%)
Sexologist (with a psychology background)	4 (15%)	4 (17%)	4 (20%)
Urologist (involved in brachytherapy/radiation therapy)	3 (11%)	2 (9%)	2 (10%)
Urologist (performing radical prostatectomies)	5 (19%)	4 (17%)	3 (15%)
*Highest academic qualification*			
Bachelor’s degree	3 (11%)	2 (9%)	2 (10%)
Honours degree	3 (11%)	2 (9%)	2 (10%)
Master’s degree	15 (56%)	13 (57%)	11 (55%)
PhD	6 (22%)	6 (26%)	5 (25%)
*Health sector*			
Government	1 (4%)	1 (4%)	1 (5%)
Private	20 (74%)	17 (74%)	15 (75%)
Private, govt and academic	2 (7%)	2 (9%)	2 (10%)
Private and academic	4 (15%)	3 (13%)	2 (10%
*Years of experience*			
< 5 years	1 (4%)		
5–10 years	4 (15%)	4 (17%)	3 (15%)
11–15 years	4 (15%)	4 (17%)	3 (15%)
16–20 years	6 (22%)	3 (13%)	3 (15%)
> 20 years	12 (44%)	12 (52%)	11 (55%)

The ratio of male to female participants were equally split throughout the rounds. Two thirds of the participants had a minimum of 15 years’ or more experience in the field. The overwhelming majority of the participants were qualified at Masters level or PhD throughout the rounds (R1: 78%, R2: 83% R3: 80%). Most participants were practicing in the private sector (74%).

### Statement agreement between participants: RD 1–3

The agreement for each statement for Rd1-3 is presented in Table 2. The results include the total responses received for each round, and percentage breakdown between the strongly agree, agree, neutral, disagree and strongly disagree options.

**Table 2:** Round 1–3 agreement results for statement 1–10

		Responses received			% Agreement				
Statement	Round	Total received	Eligible responses	Out of scope	Strongly agree	Agree	Neutral	Disagree	Strongly disagree
Statement 1	R1	27	26	1	53.8	34.6	3.8	0.0	7.7
	R2	23	23	0	78.3	13.0	4.3	4.3	0.0
	R3	–	–	–	–	–	–	–	–
Statement 2	R1	27	25	2	60.0	40.0	0.0	0.0	0.0
	R2	23	23	0	91.3	8.7	0.0	0.0	0.0
	R3	–	–	–	–	–	–	–	–
Statement 3	R1	27	27	0	59.3	37.0	0.0	3.7	0.0
	R2	23	23	0	82.6	17.4	0.0	0.0	0.0
	R3	–	–	–	–	–	–	–	–
Statement 4	R1	27	27	0	59.3	29.6	3.7	0.0	7.4
	R2	23	23	0	73.9	26.1	0.0	0.0	0.0
	R3	20	20	0	0.65	0.2	0	0	0.15
Statement 5	R1	27	27	0	44.4	33.3	0.0	14.8	7.4
	R2	23	23	0	65.2	21.7	4.3	8.7	0.0
	R3	20	20	0	0.7	0.15	0	0.1	0.05
Statement 6	R1	27	26	1	42.3	46.2	11.5	0.0	0.0
	R2	23	23	0	65.2	30.4	4.3	0.0	0.0
	R3	20	20	0	0.75	0.15	0	0	0.1
Statement 7	R1	27	27	0	48.1	37.0	7.4	7.4	0.0
	R2	23	23	0	56.5	39.1	0.0	4.3	0.0
	R3	20	20	0	0.7	0.15	0	0.1	0.05
Statement 8	R1	27	27	0	51.9	37.0	3.7	7.4	0.0
	R2	23	22	1	59.1	22.7	13.6	4.5	0.0
	R3	20	20	0	0.7	0.2	0	0	0.1
Statement 9	R1	27	27	0	59.3	25.9	3.7	11.1	0.0
	R2	23	23	0	73.9	21.7	0.0	4.3	0.0
	R3	–	–	–	–	–	–	–	–
Statement 10	R1	27	27	0	59.3	29.6	11.1	0.0	0.0
	R2	23	23	0	69.6	21.7	4.3	4.3	0.0
	R3	–	–	–	–	–	–	–	–

**Statement 1**: Please refer to box 1.

**Table:** Box 1: Agreement, statement support and statement evolution for statement 1

Agreement and statement support for statement 1				
		Round 1	Round 2	Round 3
Agreement	Number of participants	27	23	n/a
	Strongly Agree	54%	78%	n/a
Statement Support	Mean	1.60	1.23	n/a
	Standard Deviation	0.94	0.58	n/a
	Range	1–4	1–3	n/a

Evolution for Statement 1	
R1: Have you experienced involuntary loss of urine associated with sexual arousal during the last 3 months	
R2: Have you experienced involuntary leaking of urine associated with sexual arousal (besides during an orgasm)? *Arousal can be defined as the state of being sexually excited”	

***Round 1***: The word “loss” and “arousal” was found to be problematic and replaced with “leaking” and “arousal (besides during an orgasm)”. A definition of arousal was suggested and included in round 2. Adjustments were proposed related to the Likert scale that was used, and the “never to always” scale was replaced with a “very rarely to very frequently” scale. One expert (a urologist) stated that this was not a side effect, especially not after brachytherapy.

***Round 2***: It was suggested that “with or without a partner” and “with or without an erection” needed to be added to the definition of arousal. Consensus was reached*.*

**Statement 2**: Please refer to *box 2*:

**Table:** Box 2: Agreement, statement support and evolution of statement 2

Agreement and statement support for statement 2				
		Round 1	Round 2	Round 3
Agreement	Number of participants	27	23	n/a
	Strongly Agree	60%	91%	n/a
Statement Support	Mean	1.40	1.09	n/a
	Standard Deviation	0.61	0.29	n/a
	Range	1–2	1–2	n/a

Statement evolution for statement 2	
R1: Have you experienced involuntary loss of urine associated with orgasm during the last 3 months R2: Have you experienced any involuntary leaking of urine during an orgasm?	

***Round 1***: It was suggested that “with your orgasm” be replaced with “during an orgasm”, “loss” to be replaced with “leaking”. There was confusion between “orgasm” and “ejaculation”,

***Round 2***: The experts asked that a statement needed to be added that an orgasm may occur with or without ejaculation. C*onsensus was reached.*

**Statement 3**: Please refer to *box 3*:

**Table:** Box 3: Agreement, statement support and evolution of statement 3

Agreement and statement support for statement 3				
		Round 1	Round 2	Round 3
Agreement	Number of participants	27	23	n/a
	Strongly Agree	59%	83%	n/a
Statement Support	Mean	1.44	1.17	n/a
	Standard Deviation	0.58	0.39	n/a
	Range	1–3	1–2	n/a

Statement evolution for statement 3	
R1: Within the last 3 months, when you have had an orgasm, how would you characterize the intensity compared to before your prostate cancer treatment R2: Are you able to achieve an orgasm, and if yes, how would you rate the intensity of your orgasm?	

***Round 1***: In relation to the wording some experts thought that the statement implied that an orgasm was already being achieved. The first part of this statement was subsequently changed to establish whether an orgasm was being achieved. Other suggestions required an amendment to the response on the Likert scale by changing the wording from a “decrease to increase scale to “much less to much more scale”.

***Round 2***: It was suggested to swap the order of statement 2 and 3 to improve the flow of questioning. This was implemented in the final round*.* Consensus *was reached.*

**Statement 4**: Please refer to *box 4*:

**Table:** Box 4: Agreement, statement support and evolution of statement 4

Agreement and statement support for statement 4				
		Round 1	Round 2	Round 3
Agreement	Number of participants	27	23	20
	Strongly Agree	59%	74%	83%
Statement Support	Mean	1.54	1.26	1.65
	Standard Deviation	0.89	0.45	1.09
	Range	1–4	1–2	1–2

Evolution for statement 4	
R1: Within the last 3 months, have you experienced pain or discomfort when you had an orgasm R2: Have you experienced pain during an orgasm; if yes, how often does this occur; if applicable, in what area of your body do you experience the pain during an orgasm; if applicable, please describe your pain experienced during an orgasm and finally, please rate the pain you have described on the following scale (NPRS) R3: How often have you experienced pain during an orgasm; If applicable, in what area of your body have you experienced pain during an orgasm; if applicable, please describe your pain that you experienced during an orgasm; please rate the pain described above on the following scale	

***Round 1***: Suggestions were made to add a description of the area of symptoms, and to allow a way to quantify/measure the pain on a scale. This question was elaborated in round 2 to include frequency value to how often symptoms occur, a measuring capacity using the numeric pain rating scale (NPRS) and the allowance for descriptive words in the answers to allow for more detail on area of symptoms and descriptions of symptoms.

***Round 2***: A suggestion was made to replace the NPRS with the visual analogue scale. This was rejected due to the fact that sizing of the scale may change on different screens/platforms losing its reliability.

***Round 3***: A comment was made to simply state “Have you… instead of how often have you “. Another comment was made that the description of the patient’s pain would not be valuable, as it could not be used to distinguish different types of pain.

**Statement 5:** Please refer to *box 5*:

**Table:** Box 5: Agreement, statement support and evolution of statement 5

Agreement and statement support for statement 5				
		Round 1	Round 2	Round 3
Agreement	Number of participants	27	23	20
	Strongly Agree	44%	65%	70%
Statement Support	Mean	1.85	1.41	1.50
	Standard Deviation	0.95	0.71	0.89
	Range	1–4	1–4	1–4

Evolution for statement 5	
R1: Within the last 3 months, have you experienced an orgasm without ejaculating?” This statement aimed to identify anejaculation R2: When you ejaculate, has the volume of ejaculatory fluid decreased; If Yes, how much has the volume of ejaculatory fluid decreased? R3: When you ejaculate, has the volume of ejaculatory fluid decreased; If Yes, how much has the volume of ejaculatory fluid decreased?	

***Round 1***: The urologists on the panel expressed strong concern that this statement may be misleading to patients, as anejaculation is a given consequence for most post- prostatectomy patients. This was addressed in round 2, where the question was first asked whether ejaculation is able to occur. There were also concerns that some men may associate the ejaculation event as the actual orgasm event, and not be aware that an orgasm is possible without ejaculating.

***Round 2***: A comment was once again made whether prostatectomy patients would get confused, as they will not be able to ejaculate after their treatment. It was thought that the question may confuse patients and that it may leave patients concerned that their surgery was performed poorly/incorrectly.

***Round 3***: Suggestions were made to remove sections of the question. Some experts also expressed that it would be inappropriate to ask about a change in volume of ejaculate.

**Statement 6**: Please refer to *box 6*:

**Table:** Box 6: Agreement, statement support and evolution of statement 6

Agreement and statement support for statement 6				
		Round 1	Round 2	Round 3
Agreement	Number of participants	27	23	20
	Strongly Agree	44%	65%	70%
Statement Support	Mean	1.52	1.32	1.45
	Standard Deviation	0.72	0.54	0.94
	Range	1–3	1–3	1–4

Evolution of statement 6	
R1: Have you experienced one or more of the following sensory disturbances in the penis in the last 3 months? i) no disturbances, ii) sensation of cold, iii) sensation of warm, iv) felt that all or part of the penis was “asleep”, v) increased sensitivity, vi) decreased sensitivity R2: “Have you experienced any sensory changes in your penis; if yes, please indicate the sensory changes that you have experienced; if applicable, describe in your own words any other sensory changes in your penis you have experienced? i) no disturbances, ii) sensation of cold, iii) sensation of warm, iv) felt that all or part of the penis was “numb”, v) increased sensitivity, vi) decreased sensitivity R3: Have you experienced any sensory changes in your penis; if yes, please indicate the sensory changes that you have experienced; if applicable, describe in your own words any other sensory changes in your penis you have experienced? i) no disturbances, ii) sensation of cold, iii) sensation of warm, iv) felt that all or part of the penis was “numb”, v) increased sensitivity, vi) decreased sensitivity	

***Round 1***: A suggestion was made to include a section for other options that were not mentioned. The word “asleep” was queried, and suggested to be changed to “numb”, which was done.

***Round 2***: A grammar comment was made relating to the Likert scale and implemented in round 3.

***Round 3***: Suggestions were made to add the “how problematic” section to this question, similar to some of the other statement, and to remove the option to identify the type of sensation change that has occurred. These adjustments were made. Consensus was reached.

**Statement 7**: Please refer to *box 7*:

**Table:** Box 7: Agreement, statement support and evolution of statement 7

Agreement and statement support for statement 7				
		Round 1	Round 2	Round 3
Agreement	Number of participants	27	23	20
	Strongly Agree	48%	57%	70%
Statement Support	Mean	1.56	1.48	1.50
	Standard Deviation	0.75	0.59	0.89
	Range	1–3	1–3	1–3

Evolution of statement 7	
R1: Have you noticed that your penis has become shorter after your prostate cancer treatment, and if so, how much do you estimate it has changed; If you answered yes to the question above, how bothersome is it when you engage in sexual activity? R2: Has your penis become shortened in length; If yes, how problematic is it when you engage in sexual activity? R3: Has your penis become shorter in length; If yes, how problematic is it when you engage in sexual activity?	

***Round 1***: Suggestions were made to remove the options of how much the decrease in size was estimated at, and to keep the question more general. There were suggestions to change the word “bothersome” which was done in round 2.

***Round 2***: Some comments were made related to the impact of the penile shortening on self-confidence and self-image, but these were not considered for this questionnaire.

***Round 3***: Suggestions were made to add a time scale and the partners’ perspective to the question. These suggestions were not considered as the partners perspective was already invited at the start of the questionnaire, and the time scale was already included for referencing purposes.

**Statement 8**: Please refer to *box 8*:

**Table:** Box 8: Agreement, statement support and evolution of statement 8

Agreement and statement support for statement 8				
		Round 1	Round 2	Round 3
Agreement	Number of participants	27	23	20
	Strongly Agree	52%	59%	70%
Statement Support	Mean	1.54	1.37	1.50
	Standard Deviation	0.75	0.76	0.89
	Range	1–3	1–3	1–4

Evolution of statement 8	
R1: Have you noticed a different curvature of your penis after your prostate cancer treatment? If you answered yes to the question above, how bothersome is it when you engage in sexual activity? R2: Has your penis developed any new curvatures; If yes, how problematic is it when you engage in sexual activity? R3: Has your penis developed any new curvatures or bends; If yes, how problematic is it when you engage in sexual activity?	

***Round 1***: A suggestion was made to change the phrase “different curvature” to “any new curvatures”, as some minor penile curves were deemed normal. One suggestion from a urologist was to remove this question as it was not a known consequence. The same suggestions that were made to change “bothersome” in statement 7 were again made, and changes were implemented in round 2.

***Round 2***: A statement was made by a urologist that this question does not belong as it does not occur with cancer treatment. A comment was made to replace the word “curvature” with “change in shape.” This was not considered for the final round. Another comment suggested to include the word “bend” along with “curvature”, this was included in the final round.

***Round 3***: Similar comments were made to statement 7 regarding the partners perspective and time scale. A urologist on the expert panel stated that this side effect was not a consequence of PCa treatment. There was also again a suggestion to include “shape” in this question. This was not included as shape was seen as a misleading inclusion as it could mean many different things.

**Statement 9**: Please refer to *box 9*:

**Table:** Box 9: Agreement, statement support and evolution of statement 9

Agreement and statement support for statement 9				
		Round 1	Round 2	Round 3
Agreement	Number of participants	27	23	n/a
	Strongly Agree	59%	74%	n/a
Statement Support	Mean	1.50	1.30	n/a
	Standard Deviation	0.75	0.56	n/a
	Range	1–3	1–3	n/a

Evolution of statement 9	
R1: Please describe your journey with sexual dysfunction after prostate cancer treatment and/or how has sexual dysfunction impacted your life after prostate cancer R2: Describe your journey with sexual dysfunction and intimacy after prostate cancer treatment; How has this (answer above) impacted your life	

***Round 1***: Suggestions were made to split the 2 questions completely. It was also suggested to include “intimacy” with the phrase. there were many positive comments regarding the fact that this was an open-ended question, and this would give context to the symptoms.

***Round 2***: Most panellists agreed that this was an important question, but its appropriateness for inclusion in this quantitative questionnaire was questioned. Comparisons were made with other similar questionnaires that did not have open ended questions. *This question was therefore completely removed from the questionnaire.*

**Statement 10**: Please refer to *box 10*:

**Table:** Box 10: Agreement, statement support and evolution of statement 10

Agreement and statement support for statement 10				
		Round 1	Round 2	Round 3
Agreement	Number of participants	27	23	n/a
	Strongly Agree	59%	70%	n/a
Statement Support	Mean	1.33	1.26	n/a
	Standard Deviation	0.62	0.62	n/a
	Range	1–2	1–3	n/a

Evolution of statement 10	
R1: Is there anything else you want to tell us about your experience or that you think other people going through this or treating people going through this should know R2: is there anything else from your experience with your prostate cancer treatment that you want medical professionals to know; Is there anything you would like other future patients to know about?	

***Round 1***: It was suggested that the statement be split into two statements, or be rephrased as the question seemed a bit wordy.

***Round 2***: As with statement 9, most panellist agreed that this was an important question, but its appropriateness for inclusion in this quantitative questionnaire was questioned. Comparisons were made with other similar questionnaires that did not have open ended questions. This question was therefore removed from the questionnaire.

At the conclusion of the three rounds, a final screening tool was produced, and is outlined in Table 3.

**Table 3:**
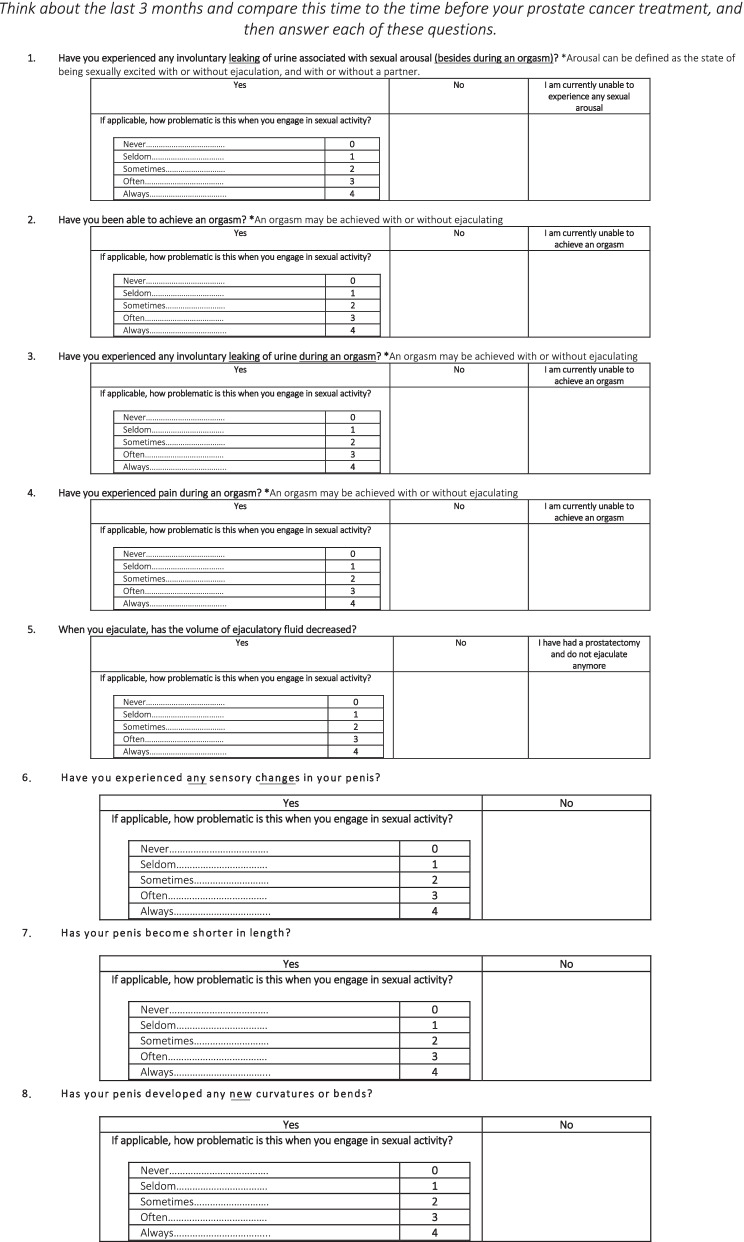
The NSSE after prostate cancer screening tool

*The full screening tool can be found as Additional file 2: Appendix 2, and gives the option for a partner of the patient to complete the questionnaire

## Discussion

The awareness of the NSSE after PCa is growing rapidly amongst health care practitioners, and with that preventative approaches are being targeted at an early stage after PCa treatment. There are currently no statistics on the prevalence rates of the NSSE after PCa on South African patients. There are however two landmark studies of the prevalence of NSSE after PCa treatment on Danish participants [12, 14]. In a 2014 study, a group of radical prostatectomy patients presented with a; 47% penile length shortening, 10% penile deformity, 38% climacturia, 25% penile sensory disturbances and 60% decreased orgasm intensity prevalence [14]. In a follow up 2017 prostate radiation (external beam radiation) study, participants presented with a; 42% penile length shortening, 12% penile deformity, 4% climacturia, 27% penile sensory disturbances, 44% decreased orgasm intensity and 11% anejaculation prevalence [12]. The scope and need to develop a screening tool to identify the evidence of a NSSE in a population of PCa survivors who have had an intervention is immense [28, 29].

A Delphi study provides an appropriate methodology to create content where there is a lack of information, incomplete knowledge or uncertainty regarding a specific topic [20, 30]. This Delphi study was conducted to establish agreement on the questions and its wording to be used for a self-administered screening tool to explore the evidence of the NSSE with a patient after their PCa treatment. A robust methodology was followed to execute this Delphi study, ensuring the quality and the consistency of the screening tool being produced. This methodology includes the composition of the expert panel, predetermining the amount of rounds, defining consensus and ensuring a short turnaround time between rounds [20].

A Delphi technique study is defined by the quality and expertise of the panel of experts that participate in the study [20]. Our expert panel included an experienced and multidisciplinary team of whom all but one (a medical sexologist) were based in South Africa. Most of these experts rendered services in private practice where the majority of early stage PCa patients are managed in South Africa due to resource limitations in the public health sector [4, 31]. These flaws in the public healthcare system have been highlighted in KwaZulu Natal where the average diagnosis of PCa is 100 days, and the vast majority diagnosis presents as advanced disease and are found in black men [32]. This trend was also seen in an earlier study looking at PCa diagnosis in in the Western Cape [5]. Sourcing an expert panel from the private sector was therefore an appropriate selection for the purposes of knowledge around early stage prostate cancer interventions in South Africa.

Three rounds of a Delphi technique study is considered optimal [30], and this Delphi technique study was completed as planned after 3 rounds, following the set out methodology [23]. Other methodological strengths of this study are that consensus was defined within the scope of 2 scenarios being that either a 75% agreement was reached or that the study rounds had expired, and the use of a Likert scale to determine participant consensus [30]. This study was also completed in 12 weeks, with a short turnaround time of 1 week between rounds, ensuring appropriate engagement from the expert panel.

Stability of consensus in this study was measured using agreement percentages and statement support parameters. Statement 2 was the most stable statement, as it had the smallest mean (1.40 and 1.09), smallest standard deviation (0.61 and 0.29) and the lowest range (1–2 and 1–2) between rounds. Statements 1 and 3 were also stable and were well supported by panellists with improvements made from round 1 to 2, and reaching consensus in round 2. While statements 4–8 all had increased in stability from round 1 to 2, they weakened from round 2 to 3 with regards to statement support from the panellists. Most comments and deliberations were made on these statements. Statements 4–8 all improved in their agreement over the 3 rounds. Statement 9 and 10 both had good stability but were removed after round 2.

Statement 5 consistently had the poorest range of statement support due to an outlier opinion of one panellist. The urologists on the panel expressed concern about the definition and wording of statement 5 (round 1) that relates to “anejaculation”. They expressed the need for unambiguity in stating that anejaculation was a given consequence after a prostatectomy and not a side effect of PCa treatment. Similar outlier opinions were noted in round 3 of statement 8, weakening the statement support in round 3 for the statement. In statement 8, one expert (urologist), repeatedly requested the removal of the Peyronies disease/penile curvature statement and argued that the disease was not a known side effect after PCa treatment. Published literature relating to Peyronies disease, however showed the presence of an abnormal penile curvature in 10% of participants in a 2014 study [14], and in 12% of the participants in a 2017 study after radiation treatment [12]. This statement was retained as part of the screening tool for statement 8. Each professional group of experts displayed specific areas of interest within the scope of the screening tool being developed. The sexologists were more interested in the details relating to the NSSE and requested for additional descriptions to further explain the sexuality aspects that may be impacted. The urologists view were biomedical and clinical, and the psychologists were concerned with the impact of the NSSE on the view of the partner of a patient. The physiotherapists and oncologists offered general comments throughout the study.

Ultimately, the experts reached 75% agreement or disagreement on 4 of the statements, and a majority agreement as per scenario 2 was reached on 4 statements. Two statements were removed and the final screening tool consisting of 8 statements was created. The argument to remove two statements (open ended questions) was successfully made by the expert panel, and their suggestion was to include this in an expansion of the screening tool or as part of a follow up conversation that would be stimulated by the screening tool.

All the experts were supportive of the development of a screening tool to screen for the NSSE’s following PCa diagnosis. The South African health care system allows for health care practitioners to work in the public sector and to spend a limited number of hours of Private remunerative work. Despite the fact that the South African health care system is still grossly inequitable, and due the severe shortages of health care personal, there is much enthusiasm to translate knowledge and interventions such as the development of a screening tool (initially for use in well-resourced private facilities) for use in the public health sector.

### Final screening tool considerations

General suggestions included the desire for the questionnaire to remain brief and uncomplicated and this was implemented in the final questionnaire. Suggestions were made to remove the subjective options describing each of the side effects, and to focus on the impact it had on sexual activity, as was the case in the initial phrasing of statements 7 and 8. These were carefully considered and subsequently implemented. All the statements in round 1 ended with “during the last 3 months”. This phrase was removed from each individual statements in round 2 and included as an instruction for patients to “think about the last 3 months and compare that to the time before your prostate cancer treatment, and then answer the question”. The final screening tool produced is outlined in Table 3.

## Study limitations

Continued commitment is required from participants who are being asked a similar question multiple times, and this may be a reason for the experts dropping out in subsequent rounds of the study. There is also no evidence of the reliability of Delphi studies if the same set of questions is presented to two different panels, and thus the success of a Delphi depends highly on the quality and experience of the expert panel. The study focuses on expected symptoms associated with current management modalities for Pea in South Africa.The findings are thus relevant to current contexts only. It will require updating with changes in treatment and would need to be tested in different populations.

## Conclusions

This study adds value in that it will assist health care practitioners to identify a variety of sexual dysfunction complications, collectively referred to as NSSE in men after PCa treatment. Currently these symptoms are often undiagnosed and remain untreated, especially in a low to middle income country such as South Africa. Consensus was reached on the statements making up the NSSE screening tool by a panel of experts. This screening tool may be applied on patients who have had treatment for early stage PCa that includes prostate surgery and prostate radiation therapies. This screening tool will need to undergo further psychometric testing to establish its validity and reliability.

## Supplementary Information

**Additional file 1: Appendix 1**. Original research statements.

**Additional file 2: Appendix 2**. NSSE after PCa Screening Tool (Full Version).

## Data Availability

The datasets used and/or analysed during the current study available from the corresponding author on reasonable request.

## Abbreviations

PCa: Prostate cancer
NSSE: Neglected Sexual Side Effects, Expanded Prostate Cancer Index
IIEF: International Index of Erectile Function
