# Supplementary material for: A modified Delphi study to identify screening items to assess neglected sexual side-effects following prostate cancer treatment
Source: BMC Urol. 2022 Mar 11;22:34. doi: 10.1186/s12894-022-00982-0 (PMC8915514; doi:10.1186/s12894-022-00982-0)
Supplement: Supplementary file 1 — Additional file 1: Appendix 1. Original research statements. [file 12894_2022_982_MOESM1_ESM.docx]

**Appendix 1**

Original Research Statements

Round 1 Delphi Technique Study

**The expert panel were asked to rate the appropriateness of statement 1-10 and their accompanying answers by rating it on a Likert scale:**

**The Likert scale options were:**

*You strongly disagree that the item is appropriate*

*You disagree that the item is appropriate*

*You neither agree or disagree that the item is appropriate*

*You agree that the item is appropriate*

*You strongly disagree that the item is appropriate*

*This item falls outside the scope of my expertise. I am unable to advise on this item*

The option was also provided to comments or suggestions for changes from the expert panel

**Statement 1-10**

**Statement 1:** Please indicate how appropriate you think the following question and possible answers are with regards to sexual arousal urinary incontinence.

| **Have you experienced involuntary loss of urine associated with sexual arousal during the last 3 months?** | **Mark**  **X** |
| --- | --- |
| No arousal possible |  |
| Almost never or never |  |
| A few times (less than half) |  |
| Sometimes (about half the time) |  |
| *Most of the time (more than half the time)* |  |
| Almost always or always |  |

**Statement 2:** Please choose how appropriate you think the following question and possible answers are with regards to Climacturia/ Orgasm Associated Incontinence

| **Have you experienced involuntary loss of urine associated with your orgasms during the last 3 months?** |  |
| --- | --- |
| No Orgasms |  |
| Almost never or never |  |
| A few times (less than half) |  |
| Sometimes (about half the time) |  |
| *Most of the time (more than half the time)* |  |
| Almost always or always |  |

**Statement 3:** Please choose how appropriate you think the following question and possible answers are with regards to Changes in Orgasm

| **Within the last 3 months, when you have had an orgasm, how would you characterize the intensity compared to before your prostate cancer treatment?** | **Mark x** |
| --- | --- |
| No orgasm (you have not been able to achieve an orgasm) |  |
| Decreased intensity |  |
| Unchanged intensity |  |
| Increase intensity |  |

**Statement 4:** Please choose how appropriate you think the following question and possible answers are with regards to Pain Associated with Orgasm

| **Within the last 3 months, have you experienced pain or discomfort when you have had an orgasm?** | **Mark x** |
| --- | --- |
| No orgasms |  |
| Almost never or never |  |
| A few times (less than half) |  |
| Sometimes (about half the time) |  |
| Most of the time (more than half the time) |  |
| Almost always or always |  |

**Statement 5:** Please choose how appropriate you think the following question and answers are with regards to Anejaculation.

| **Within the last 3 months, have you experienced an orgasm without ejaculating?** | **Mark x** |
| --- | --- |
| No orgasms |  |
| Almost never or never (you are ejaculating as before the treatment) |  |
| A few times (less than half) |  |
| Sometimes (about half the time) |  |
| Most of the time (more than half the time) |  |
| Almost always or always |  |

**Statement 6:** Please choose how appropriate you think the following question and possible answers are with regards to Penile Sensation Changes.

| **Have you experienced one or more of the following sensory disturbances in the penis in the last 3 months?** | **Mark x** |
| --- | --- |
| No disturbances |  |
| Sensation of cold |  |
| Sensation of warm |  |
| Felt that all or part of the penis was “asleep” |  |
| Increased sensitivity |  |
| Decreased sensitivity |  |

**Statement 7:** Please choose how appropriate you think the following question and possible answers are with regards to Changes in Penile Size.

| **Have you noticed that your penis has become shorter after your prostate cancer treatment, and if so, how much do you estimate it has changed?** | **Mark x** |
| --- | --- |
| No change |  |
| 0–1 cm |  |
| 1–3 cm |  |
| 3–5 cm, |  |
| More than 5 cm. |  |

| **If you answered yes to the question above, how bothersome is it when you engage in sexual activity?** | **Mark x** |
| --- | --- |
| Not bothersome at all |  |
| Slightly bothersome |  |
| Moderately bothersome |  |
| Quite a bit bothersome |  |
| Extremely bothersome |  |

**Statement 8:** Please choose how appropriate you think the following question and possible answers are with regards to Peyronie-like disease/Penile Curvature**.**

| **Have you noticed a different curvature of your penis after your prostate cancer treatment?** | **Mark x** |
| --- | --- |
| Yes |  |
| No |  |

| **If you answered yes to the question above, how bothersome is it when engaging in sexual activity?** | **Mark x** |
| --- | --- |
| Not bothersome at all |  |
| Slightly bothersome |  |
| Moderately bothersome |  |
| Quite a bit bothersome |  |
| Extremely bothersome |  |

**Statement 9:** Please choose how appropriate you think the following question is with regards to the following open-ended question:

**Experiences:** Please answer the following questions in your own words:

Please describe your journey with sexual dysfunction after prostate cancer treatment and/or how has sexual dysfunction impacted your life after prostate cancer treatment?

**Statement 10:** Please choose how appropriate you think the following question is with regards to the following open-ended question.

**Experiences:** Please answer the following questions in your own words:

Is there anything else you want to tell us about your experience or that you think other people going through this or treating people going through this should know?
