# Supplementary material for: A modified Delphi study to identify screening items to assess neglected sexual side-effects following prostate cancer treatment
Source: BMC Urol. 2022 Mar 11;22:34. doi: 10.1186/s12894-022-00982-0 (PMC8915514; doi:10.1186/s12894-022-00982-0)
Supplement: Supplementary file 2 — Additional file 2: Appendix 2. NSSE after PCa Screening Tool (Full Version). [file 12894_2022_982_MOESM2_ESM.docx]

**Appendix 2**

**The NSSE after PCa Screening Tool (Additional Demographic Information)**

| **Who is completing this questionnaire?** | **Mark x** |
| --- | --- |
| You are completing this questionnaire by yourself |  |
| You are completing this questionnaire with your partner/spouse |  |
| You are completing this questionnaire on behalf of your partner, based on their experience. (*Please complete the information based on our partner.)* |  |

**About you:**

**How old are you:**

| **Race** | **Mark x** |
| --- | --- |
| *White* |  |
| Black |  |
| Coloured |  |
| Asian |  |
| Other (please specify) …………………………. |  |

| **Are you on medication to treat Hypertension?** | **Mark x** |
| --- | --- |
| Yes |  |
| No |  |
| Specify if YES (name and dose) | |

| **Are you on medication to treat Depression?** | **Mark x** |
| --- | --- |
| Yes |  |
| No |  |
| Specify if YES (name and dose) | |

| **Did you receive your prostate cancer management in the private or government sector?** | **Mark x** |
| --- | --- |
| Private sector |  |
| Government Sector |  |
| Both Private and Government Sector (Please elaborate) | |

| **What was the stage of the prostate cancer when you were initially diagnosed?** | **Mark x** |
| --- | --- |
| Stage 1 |  |
| Stage 2 |  |
| Other (Please specify) | |

| **Indicate the management you have received and indicate how long ago the treatment was done.** | **Mark x** |
| --- | --- |
| Robotic Prostatectomy |  |
| If so, how long ago did you finish your treatment | |
| Laparoscopic Prostatectomy |  |
| If so, how long ago did you finish your treatment | |
| Open prostatectomy |  |
| If so, how long ago did you finish your treatment | |
| Radiation (External Beam Radiation) |  |
| If so, how long ago did you finish your treatment | |
| Radiation (Brachytherapy) |  |
| If so, how long ago did you finish your treatment | |
| Other (please specify): |  |
| If so, how long ago did you finish your treatment | |

| **Are you currently sexually active?** | **Mark x** |
| --- | --- |
| Yes, with a partner |  |
| Yes, but without a partner |  |
| No, not at all |  |
| **Are you on medication to treat Erectile Dysfunction?** | **Mark x** |
| Yes |  |
| No |  |
| Specify if YES (name and dose) | |

| **What is your perceived effectiveness of the above mention drug/drugs (choose one)?** | **Mark x** |
| --- | --- |
| Not effective |  |
| Somewhat effective |  |
| Effective |  |
| Very effective |  |
| *Extremely effective* |  |

| **Are you currently using a vacuum erectile device?** | **Mark x** |
| --- | --- |
| Yes |  |
| No |  |

| **If yes, what is your perceived effectiveness of the vacuum erectile device?** | **Mark x** |
| --- | --- |
| Not effective |  |
| Somewhat effective |  |
| Effective |  |
| Very effective |  |
| *Extremely effective* |  |

| **Are you currently using a penile prosthesis?** | **Mark x** |
| --- | --- |
| Yes |  |
| No |  |

| **If yes, what is your perceived effectiveness of the penile prosthesis?** | **Mark x** |
| --- | --- |
| Not effective |  |
| Somewhat effective |  |
| Effective |  |
| Very effective |  |
| *Extremely effective* |  |

**The Neglected Sexual Side Effects After Prostate Cancer Screening Tool**

*Think about the last 3 months and compare this time to the time before your prostate cancer treatment, and then answer each of these questions.*

1. **Have you experienced any involuntary leaking of urine associated with sexual arousal (besides during an orgasm)?** *Arousal can be defined as the state of being sexually excited with or without ejaculation, and with or without a partner.

| **Yes** | **No** | **I am currently unable to experience any sexual arousal** |
| --- | --- | --- |
| **If applicable, how problematic is this when you engage in sexual activity?**   \| Never………………………………. \| **0** \| \| --- \| --- \| \| Seldom……………………………. \| **1** \| \| Sometimes………………………. \| **2** \| \| Often………………………………. \| **3** \| \| Always……………………………... \| **4** \| |  |  |

1. **Have you been able to achieve an orgasm? ***An orgasm may be achieved with or without ejaculating

| **Yes** | **No** | **I am currently unable to achieve an orgasm** |
| --- | --- | --- |
| **If applicable, how problematic is this when you engage in sexual activity?**   \| Never………………………………. \| **0** \| \| --- \| --- \| \| Seldom……………………………. \| **1** \| \| Sometimes………………………. \| **2** \| \| Often………………………………. \| **3** \| \| Always……………………………... \| **4** \| |  |  |

1. **Have you experienced any involuntary leaking of urine during an orgasm? ***An orgasm may be achieved with or without ejaculating

| **Yes** | **No** | **I am currently unable to achieve an orgasm** |
| --- | --- | --- |
| **If applicable, how problematic is this when you engage in sexual activity?**   \| Never………………………………. \| **0** \| \| --- \| --- \| \| Seldom……………………………. \| **1** \| \| Sometimes………………………. \| **2** \| \| Often………………………………. \| **3** \| \| Always……………………………... \| **4** \| |  |  |

1. **Have you experienced pain during an orgasm? ***An orgasm may be achieved with or without ejaculating

| **Yes** | **No** | **I am currently unable to achieve an orgasm** |
| --- | --- | --- |
| **If applicable, how problematic is this when you engage in sexual activity?**   \| Never………………………………. \| **0** \| \| --- \| --- \| \| Seldom……………………………. \| **1** \| \| Sometimes………………………. \| **2** \| \| Often………………………………. \| **3** \| \| Always……………………………... \| **4** \| |  |  |

1. **When you ejaculate, has the volume of ejaculatory fluid decreased?**

| **Yes** | **No** | **I have had a prostatectomy and do not ejaculate anymore** |
| --- | --- | --- |
| **If applicable, how problematic is this when you engage in sexual activity?**   \| Never………………………………. \| **0** \| \| --- \| --- \| \| Seldom……………………………. \| **1** \| \| Sometimes………………………. \| **2** \| \| Often………………………………. \| **3** \| \| Always……………………………... \| **4** \| |  |  |

1. **Have you experienced any sensory changes in your penis?**

| **Yes** | **No** |
| --- | --- |
| **If applicable, how problematic is this when you engage in sexual activity?**   \| Never………………………………. \| **0** \| \| --- \| --- \| \| Seldom……………………………. \| **1** \| \| Sometimes………………………. \| **2** \| \| Often………………………………. \| **3** \| \| Always……………………………... \| **4** \| |  |

1. **Has your penis become shorter in length?**

| **Yes** | **No** |
| --- | --- |
| **If applicable, how problematic is this when you engage in sexual activity?**   \| Never………………………………. \| **0** \| \| --- \| --- \| \| Seldom……………………………. \| **1** \| \| Sometimes………………………. \| **2** \| \| Often………………………………. \| **3** \| \| Always……………………………... \| **4** \| |  |

1. **Has your penis developed any new curvatures or bends?**

| **Yes** | **No** |
| --- | --- |
| **If applicable, how problematic is this when you engage in sexual activity?**   \| Never………………………………. \| **0** \| \| --- \| --- \| \| Seldom……………………………. \| **1** \| \| Sometimes………………………. \| **2** \| \| Often………………………………. \| **3** \| \| Always……………………………... \| **4** \| |  |
